# Cognitive Inflexibility Predicts Extremist Attitudes

**DOI:** 10.3389/fpsyg.2019.00989

**Published:** 2019-05-07

**Authors:** Leor Zmigrod, Peter Jason Rentfrow, Trevor W. Robbins

**Affiliations:** ^1^Department of Psychology, University of Cambridge, Cambridge, United Kingdom; ^2^Behavioural and Clinical Neuroscience Institute, University of Cambridge, Cambridge, United Kingdom

**Keywords:** ideology, cognitive flexibility, extremism, intergroup attitudes, identity fusion

## Abstract

Research into the roots of ideological extremism has traditionally focused on the social, economic, and demographic factors that make people vulnerable to adopting hostile attitudes toward outgroups. However, there is insufficient empirical work on individual differences in implicit cognition and information processing styles that amplify an individual’s susceptibility to endorsing violence to protect an ideological cause or group. Here we present original evidence that objectively assessed cognitive inflexibility predicts extremist attitudes, including a willingness to harm others, and sacrifice one’s life for the group. Across two samples (*N* = 1,047) from the United Kingdom and United States, structural equation models demonstrated that cognitive inflexibility predicted endorsement of violence to protect the national ingroup, which in turn predicted a willingness to die for the group. These statistical models accounted for an average of 31.4% of the variance in willingness to die for the group, after accounting for demographic variables. Furthermore, cognitive inflexibility was related to greater confidence in the decision to sacrifice one’s life in an ingroup trolley problem scenario. Analysis of participants’ performance on the cognitive tasks revealed that cognitive rigidity – distinctly from other aspects of cognition – was specifically implicated as a cognitive antecedent of extremist attitudes. Implications for the study of radicalization and identity fusion through a neurocognitive lens are discussed.

## Introduction

Psychologists have sought to identify the psychological underpinnings of authoritarianism, ethnocentrism, and xenophobia since the beginning of the 20th century. One prominent hypothesis developed in the 1940s proposed that ideological rigidity is rooted in mental rigidity. Specifically, it was suggested that “one of the characteristics of ethnocentric thinking is a rigidity and inflexibility of the thinking process” (Rokeach, 1948, p. 259) and “general rigidity and intolerance…serve as primary sources of the specific phenomena in the prejudice area” (Hartley, 1946). This hypothesis emerges from the notion that extreme group identities and ideologies are often characterized by a tendency to categorize the world and people in an inflexible and essentialist manner (Tajfel and Turner, 1979; Brewer, 1999). Consequently, individuals with a more categorical, inflexible thinking style may tend to adhere to ideologies in a stricter or more extreme fashion. Adorno and colleagues’ (Adorno et al., 1950) pivotal book, *The Authoritarian Personality*, further developed these ideas by providing empirical support to the hypothesis that prejudice is tightly linked to rigidity and intolerance of ambiguity. Indeed, Else Frenkel-Brunswick (one of the authors of *The Authoritarian Personality*) already noted in 1949 that children who scored highly on prejudice measures exhibited greater rigidity on arithmetic and perceptual tasks than children who scored low on prejudice. The hypothesis that ideological rigidity originates from psychological rigidity thus sparked a rich line of research in political psychology, under the assumption – well-articulated by Gordon Allport in *The Nature of Prejudice* – that “the style of thinking that is characteristic of prejudice is a reflection, by and large, of the prejudiced person’s way of thinking about *anything*” (Allport, 1954, p. 400; emphasis in original; cited in Roets and Van Hiel, 2011).

While the original inflexibility hypothesis was primarily concerned with ethnocentrism and intolerance, modern political psychology has focused on the relationship between psychological rigidity and politically right-wing attitudes (Jost et al., 2003; Van Hiel et al., 2010, 2016), rather than with ideological intergroup attitudes more generally. Nonetheless, ideological commitment can be evident in one’s nationalistic attachment (Mummendey et al., 2001; Zmigrod et al., 2018a), religious doctrine (Besta et al., 2014; Fredman et al., 2017), political attitudes (Jost, 2017), or even deep loyalty to a sports team (Xiao and Van Bavel, 2012). Such ideological attachments can often motivate individuals to endorse the pursuit of hostile actions against those that appear to threaten the group and its unifying cause. This is evident in religious fundamentalism, xenophobic political groups, and football hooliganism. Ideological extremism consists of “the justification of intergroup violence and demand for sacrifice in defense of the group” (McCauley and Moskalenko, 2008), and so it is possible to investigate its precursors in the general population by assessing individuals’ willingness to fight and sacrifice for an ideological ingroup.

Notably, there has been little integration of the inflexibility hypothesis into the modern understanding of extremist behavior. While a rich literature has emerged on the role of interpersonal and identity processes in shaping support for extreme pro-group actions (Kruglanski et al., 2014, 2017; Swann et al., 2014, 2015), the possible *cognitive* antecedents of extremist attitudes are still not well understood. Several key questions are yet to be answered. For instance, how do information processing and attentional styles shape individuals’ susceptibility to adopting extremist beliefs? Do changes in cognitive processing result in altered evaluation of socio-political arguments? And does engagement with an extremist ideology lead to changes in general cognition?

While the role of *cognitive processes* in extremism has been overlooked, the contribution of *psychological motivations* has been discussed at length. The distinction is important: cognition refers to how information is attended to, processed, and evaluated, whereas motivation reflects the contributions of reward and punishment to individuals’ initiation, persistence, and termination of particular behaviors. One theoretical framework that has explored the psychological motivations behind radicalization is *significance quest theory* (SQT; Kruglanski and Fishman, 2009; Kruglanski et al., 2013, 2014, 2017). SQT posits that extreme behaviors reflect means of obtaining or restoring an individual’s experience of personal significance and efficacy (Webber et al., 2017). Experiencing a loss of significance due to personal or collective humiliation can therefore catalyze individuals to adopt extreme ideologies that offer clear means of restoring meaning and motivational focus. In particular, Kruglanski and colleagues have identified *need for cognitive closure* (NCC), a motivational state in which individuals seek unambiguous and absolute answers, as a mediator between loss of significance and extremism (Webber et al., 2017). Dispositional high need for cognitive closure also predicts outgroup discrimination (Brizi et al., 2016), prejudice (Van Hiel et al., 2004; Roets and Van Hiel, 2006, 2011; Onraet et al., 2011), implicit racism (Cunningham et al., 2004), and religious fundamentalism (Brandt and Reyna, 2010).

Webster and Kruglanski (1997) noted the differences between NCC and the rigidity constructs discussed in the early theories of Rokeach (1948), Frenkel-Brunswik (1949), and others. As Webster and Kruglanski point out, NCC and rigidity are similar in their “tendency to be rejecting and impervious in regard to new ideas or experiences” (Webster and Kruglanski, 1997, p. 138) but differ in that the early theorists focused on “*cognitive* rather than *motivational*” (Webster and Kruglanski, 1997, p. 138) processes. The present investigation thereby re-focuses the empirical lens back to *cognitive* traits, rather than the motivational processes discussed by Kruglanski and colleagues, and operationalizes them through the modern methodologies of cognitive psychology.

The current study had three objectives. Firstly, it aimed to test the original hypothesis of a link between cognitive inflexibility and rigid ingroup mentality using objective, behavioral measures of cognitive flexibility. This is particularly important given that a majority of studies rely on self-reported measures of inflexibility, and Van Hiel et al. (2016) recent found that operationalizing cognitive style using self-report measures rather than behavioral assessments can inflate effect sizes. Cognitive inflexibility is defined as the inability to switch between modes of thinking and hence a difficulty to adapt to changing rules or categories (Cools and Robbins, 2004). Cognitive flexibility was objectively assessed using three validated cognitive tasks that tap into implicit cognitive tendencies to categorize information and rules in a flexible vs. more rigid fashion: the Wisconsin Card Sorting Test (WCST), Remote Associates Test (RAT), and Alternative Uses Test (AUT, in Study 2 only). The classic WCST (Grant and Berg, 1948) measures how easily individuals adapt to changes in newly learnt rules and reward contingencies and the extent to which individuals can switch between categories when it is disadvantageous to persist with a previously rewarded category. High scores indicate a flexible cognitive processing style. The RAT (Mednick, 1968) measures individuals’ ability to generate semantic connections between remote concepts. For instance, participants are shown three remotely connected words (e.g., *worm, shelf*, and *end*) and asked to find the compound word that links them (e.g., *book*). The RAT therefore indicates the extent to which participants’ semantic networks tend to categorize concepts more loosely – which would aid detection of remote conceptual connections – or rigidly, which would render such retrieval challenging (Zmigrod and Zmigrod, 2016). The AUT (Guilford, 1967) requires that participants provide as many conventional and unconventional uses to common objects, such as a brick or a hairpin, and thereby assesses four cognitive dimensions, including flexibility. Flexibility is quantified as the number of distinct conceptual categories into which a participant’s set of responses can be divided. It has been used as a measure of cognitive flexibility in multiple behavioral and neuroimaging studies (e.g., Roberts et al., 2017; Zmigrod et al., 2018b, 2019).

The second aim was to test the hypothesis that cognitive inflexibility is related to ideological thinking in the context of extremist attitudes, as operationalized by a willingness to fight, and die for the national ingroup (Swann et al., 2010, 2012; Fredman et al., 2015). This differs from past attempts to relate cognitive flexibility, cognitive ability, or need for cognitive closure to right-wing attitudes or general prejudice (e.g., Sidanius, 1985; Jost et al., 2003; Van Hiel et al., 2010, 2016; Roets and Van Hiel, 2011; Hodson and Busseri, 2012; Dhont and Hodson, 2014). Examining the relationship between cognitive inflexibility and extremist intergroup attitudes therefore constitutes a novel addition to the literature.

Thirdly, the study tested whether the relationship between cognitive rigidity and ideological attachment ultimately predicts willingness to sacrifice one’s life to protect the national ingroup. Recent research has demonstrated that close attachment and tight relational ties to a group promote individuals’ willingness to self-sacrifice on behalf of the group (Swann et al., 2009, 2015). Consequently, willingness to fight for the group was treated as a separate, antecedent construct to willingness to die for the group. To the best of our knowledge, the role of cognitive styles in such self-sacrificing tendencies has not been empirically studied.

## Study 1 – United Kingdom Sample

### Introduction

Building on the theoretical and empirical work outlined above, three specific hypotheses guided Study 1:

*H1*: Cognitive inflexibility is related to heightened willingness to fight for one’s ingroup against outgroups.
*H2*: Cognitive inflexibility predicts willingness to sacrifice oneself in favor of one’s ingroup through its effect on ideological attachment, i.e., willingness to fight for one’s ingroup.
*H3*: Cognitive inflexibility predicts greater ideological conviction in one’s willingness to sacrifice oneself in favor of the ingroup.

### Materials and Methods

#### Participants and Procedure

We sought to recruit 286 participants via Prolific Academic to achieve greater than 80% power to detect a medium effect of *r* = 0.20 (according to a meta-analysis by Gignac and Szodorai (2016) at α = 0.01 in our primary analyses of correlations between the cognitive variables and the endorsement of extreme pro-group actions, calculated using the pwr package in R (Champely et al., 2018). We oversampled by 7% and recruited 305 participants, 1 of which was excluded due to providing incomplete responses, yielding a total sample of 304 participants. Participants were redirected from Prolific Academic to an online survey hosted by Qualtrics Survey Software for completion of all the self-reported items and the RAT, and later redirected again to Inquisit 5 by Millisecond Software in order to temporarily download software that allows for accurate measure of performance and reaction times in the WCST. Participants were asked about demographic variables such as age, ethnicity, gender, and educational attainment. In the United Kingdom sample, the average age was 38.02 (*SD* = 13.51, range = 18–72), and 47.0% of participants were female (see Supplementary Table S1 for additional demographic characteristics). The research was conducted with the ethical approval of Cambridge University’s Department of Psychology Research Ethics Committee.

#### Wisconsin Card Sorting Test (WCST)

The WCST (Grant and Berg, 1948) was administered with Inquisit 5 by Millisecond Software in standard fashion (Heaton, 1981). Participants are presented with four key cards and a deck of response cards that vary on three dimensions (color, shape, and number of geometric figures) and are asked to match a fifth card from the sequentially presented response cards to one of the four key cards. Participants need to identify the correct classification rule (out of three potential rules: matching by color, shape, or number) according to the feedback they receive after each trial. They are informed that the classification rule may change without warning, and indeed the rule alternates after participants correctly respond to ten consecutive trials, requiring a flexible set shift. The task ends after participants complete six categories (twice for each of the three rules) or after 128 trials. To index participants’ performance, the accuracy rate was computed.

#### Remote Associates Test (RAT)

The RAT (Mednick, 1968) consisted of 20 compound remote associate problems, in which participants are presented with three cue words (e.g., *cottage, swiss*, and *cake*), and must generate the compound word solution that connects these three words (e.g., *cheese*). Items of varying difficulty levels were selected from established remote associate problems (Bowden and Jung-Beeman, 2003). Participants were given 20 seconds to respond to each item.

#### Willingness to Fight for the Group

Willingness to fight for the group was measured using Swann and colleagues’ validated 5-item measure (Cronbach’s α = 0.82): (a) “I would fight someone physically threatening another British person,” (b) “I would fight someone insulting or making fun of the United Kingdom as a whole,” (c) “I would help others get revenge on someone who insulted the United Kingdom,” (d) “Hurting other people is acceptable if it means protecting the United Kingdom,” and (e) “I’d do anything to protect the United Kingdom.” All items were presented on a 7-point scale ranging from *totally disagree* to *totally agree*.

#### Willingness to Die for the Group

Willingness to die for the group was measured using Swann et al. (2010) item presented on a 7- point scale ranging from *totally disagree* to *totally agree*: “I would sacrifice my life if it saved another group member’s life.”

#### Trolley Problem – Willingness to Jump to One’s Death to Save the Group

Participants were presented with the following variation of the footbridge dilemma, inspired by that presented by Swann et al. (2010): “*Imagine that a runaway trolley is about to crush and kill 5 British people. You have the opportunity to jump from a bridge into the trolley’s path and save all 5 British people. Would you: (a) let the trolley crush the 5 British people and save your own life, OR (b) save the 5 British people and sacrifice your own life?”* Participants were then asked to choose between the two options.

In this sample, after indicating whether they would either sacrifice themselves for the 5 fellow ingroup members or save their own life by letting the trolley crush and kill the 5 ingroup members, they were asked to indicate on a scale of 1–100 their certainty that they would behave as they originally indicated. Specifically, they were asked: “How certain are you that you would let the trolley crush the 5 British people and save your own life/save the 5 British people and sacrifice your own life?”

### Results

#### H1: Is Cognitive Inflexibility Correlated With Extremist Attitudes?

Performance on the WCST and RAT was analyzed in relation to individuals’ support for extreme pro-group actions. Correlational analysis revealed significant negative correlations between willingness to fight and kill outgroup members to protect the ingroup and cognitive flexibility across both tasks (Table 1).

**Table 1:** Pearson’s correlations between cognitive flexibility measures and ideological attitudes across the two samples.

	RAT accuracy rate	WCST accuracy rate	AUT flexibility score
**Willingness to fight for the group**			
United Kingdom sample	*r* = -0.241^∗∗∗^ *p* < 0.001	*r* = -0.216^∗∗^ *p =* 0.002	–
United States sample	*r* = -0.231^∗∗∗^ *p* < 0.001	*r* = -0.133^∗^ *p =* 0.010	*r* = -0.200^∗∗∗^ *p < 0*.001
**Willingness to die for the group**			
United Kingdom sample	*r* = -0.207^∗∗∗^ *p* = 0.001	*r* = -0.039 *p* = 0.587	–
United States sample	*r* = -0.073 *p* = 0.069	*r* = -0.005 *p* = 0.917	*r* = -0.084^∗^ *p* = 0.026
**Identity fusion**			
United States sample	*r =* -0.101^∗^ *p* = 0.010	*r* = -0.031 *p* = 0.531	*r* = -0.049 *p* = 0.191

*^∗^p < 0.05, ^∗∗^*p* < 0.01, ^∗∗∗^*p* < 0.001.*

#### H2: Does Cognitive Inflexibility Predict Violence Endorsement Against Outgroups and Self-Sacrificial Tendencies?

To develop a more comprehensive understanding of how cognitive flexibility contributes to extremist attitudes, we specified a type of structural equation model called “path models.” This allowed us to examine the extent to which cognitive inflexibility predicts willingness to fight for the group and whether this in turn predicts willingness to die for the group. We used the R package Lavaan (Rosseel, 2012, 2017) to compute these models. A three-level model was fitted to the data, in which Level 1 included the two cognitive flexibility measures (WCST and RAT), Level 2 consisted of willingness to kill and fight for the group, and Level 3 reflected individuals’ willingness to sacrifice themselves for their national group. In this model specification, the cognitive flexibility measures in Level 1 directly predicted the variables in Level 2, which in turn predicted the self-sacrificial attitudes in Level 3. Residual covariances were allowed within, but not between, levels. All analyses controlled for age, gender, and educational attainment, and residual covariances were allowed between these demographic variables.

We first tested a model in which willingness to self-sacrifice for the group (Level 3) was directly predicted by the willingness to fight for the group in Level 2 as well as the cognitive flexibility variables in Level 1. This model had good fit to the data (χ^2^ = 17.692, df = 6, *p* = 0.007, *n* = 304, RMSEA = 0.080 [0.040, 0.123], SRMR = 0.024, CFI = 0.920, Yuan-Bentler scaling correction factor = 1.075). This model, in which all pathways between the cognitive flexibility measures and willingness to self-sacrifice were estimated freely, was compared to a more parsimonious model, in which all pathways between Level 1 and Level 3 were constrained to equal 0. This parsimonious model reflects the assumption that the effect of cognitive flexibility on willingness to self-sacrifice is mediated completely via ideological attachment in Level 2. This model also had good fit to the data (χ^2^ = 20.431, df = 8, *p* = 0.009, *n* = 304, RMSEA = 0.071 [0.035, 0.109], SRMR = 0.056, CFI = 0.915, Yuan-Bentler scaling correction factor = 1.063). A likelihood ratio test showed no significant difference between the two models in terms of model fit (Δχ^2^ = 2.7014, Δdf = 2, *p* = 0.2591), suggesting that the more parsimonious model – in which there are no direct pathways between Level 1 and Level 3, and so the variables in Level 2 possess a full mediatory role – is preferred.

This model accounted for 14.5% of the variance in willingness to fight for the group and 33.5% of the variance in participants’ willingness to die for the national group. While WCST was not a significant predictor of willingness to fight for the group, its contribution approached significance (*p* = 0.10). To probe this further, an SEM was fitted in which the pathway between willingness to fight for the group and WCST was estimated freely while the pathway between willingness to fight for the group and RAT was constrained to equal 0. This suggested that WCST is predictive of willingness to fight for the group (unstandardized estimate = -5.629, SE = 2.890, standardized estimate = -0.149, *p* = 0.051) but its variance is largely accounted for by RAT, such that WCST performance is associated with willingness to fight for the ingroup but does not predict above and beyond RAT performance.

Additionally, we tested whether this model structure would also predict participants’ behavior in the trolley dilemma, however, the model was not an adequate fit to the data [χ^2^ = 16.573, df = 8, *p* = 0.035, *n* = 304, RMSEA = 0.078 (0.020, 0.131), SRMR = 0.056, CFI = 0.815], primarily because willingness to fight for the group did not predict trolley dilemma behavior (*p* = 0.884).

#### H3: Is Cognitive Inflexibility Related to Conviction in Self Sacrifice?

**FIGURE 1:**
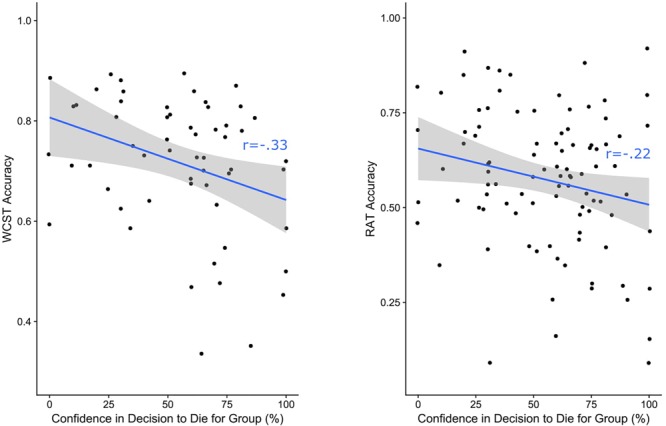
Correlations between cognitive flexibility (WCST and RAT) and participants’ confidence in their decision to sacrifice themselves for the group in a hypothetical ingroup trolley dilemma.

We further examined the relationship between cognitive flexibility and willingness to self-sacrifice for members of a national ingroup by analyzing participants’ behavior in the trolley dilemma. While there were no differences in the cognitive flexibility of individuals who indicated that they would self-sacrifice vs. save themselves in the trolley dilemma in terms of WCST [*F*(1,205) = 0.098, *p* = 0.754] or RAT [*F*(1,294) = 0.815, *p* = 0.367], the level of confidence in their decision to self-sacrifice was related to cognitive flexibility. Specifically, amongst participants who indicated that they would sacrifice themselves to save 5 fellow British citizens, greater confidence in the decision to self-sacrifice was related to reduced cognitive flexibility in the WCST (*r* = -0.333, *p* = 0.011) and RAT (*r* = -0.217, *p* = 0.034). Consequently, individuals with heightened conviction that they would die for the group exhibited reduced cognitive flexibility relative to individuals who were less confident in their decision to self-sacrifice (see Figure 1). Notably, amongst participants who chose to save themselves in the trolley dilemma, there were no significant correlations between confidence in the decision to save themselves and cognitive flexibility (WCST: *r* = -0.037, *p* = 0.659; RAT: *r* = -0.087, *p* = 0.230). This suggest that cognitive inflexibility is linked specifically to conviction in self sacrifice.

### Interim Discussion

**FIGURE 2:**
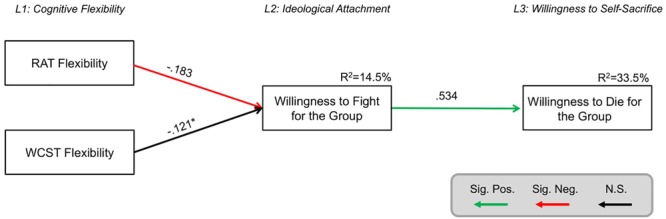
Structural equation model predicting willingness to die for the group. All parameters shown are fully standardized. For unstandardized estimates, SEs, and confidence intervals, see Supplementary Data. Significant parameter estimates are shown in red and green bolded lines. Residual covariances between variables on the same level are allowed, but not shown for simplicity. Model controls for demographic variables (age, gender, and educational attainment). Significance level was *p* < 0.05. ^∗^*p* = 0.10. L1, level 1; L2, level 2; and L3, level 3. Figure design was inspired by Kievit et al. (2016).

The results of Study 1 corroborate the three main hypotheses and offer nuanced insights. Regarding *H1*, cognitive inflexibility on both WCST and RAT was related to heightened willingness to endorse violence against outgroups in order to protect the ingroup (Table 1). Correlational analyses demonstrated that willingness to sacrifice one’s life for the ingroup was negatively correlated with RAT performance but not WCST performance (Table 1). Structural equation models illustrated that the two cognitive flexibility measures simultaneously predicted willingness to endorse violence against outgroups, and this in turn predicted willingness to sacrifice one’s life to save ingroup members (Figure 2), corroborating *H2*. Lastly, behavior on the hypothetical ingroup trolley dilemma revealed that while there were no differences in cognitive inflexibility according to whether participants opted for self-sacrifice or self-preservation, greater confidence in the decision to self-sacrifice was related to cognitive inflexibility (Figure 1), supporting *H3*. This relationship was specific to the decision to self-sacrifice and was absent in the decision to self-preserve, suggesting that self-sacrificial tendencies possess unique cognitive correlates.

## Study 2 – United States Sample

### Introduction

The objective of Study 2 was to replicate the findings of Study 1 and extend them to incorporate ideas and constructs from identity fusion theory (Swann et al., 2009, 2012; Swann and Buhrmester, 2015), which posits that a feeling of oneness and identity fusion with the group facilitate pro-group actions and self-sacrifice. Moreover, Study 2 aimed to investigate whether endorsement of extreme pro-group actions was specifically related to cognitive flexibility, or rather implicated several cognitive traits. To this end, we incorporated an additional measure, the Alternative Uses Test, which indexes cognitive flexibility as well as other cognitive constructs such as fluency, elaboration, and originality. Study 2 therefore sought to replicate *H1* and *H2*, outlined in Study 1, and investigate two additional hypotheses:

*H4*: Cognitive inflexibility is specifically implicated in endorsement of violence against outgroups. Other cognitive indices will be unrelated to willingness to fight outgroup members to protect the ingroup.
*H5*: Cognitive inflexibility will predict self-sacrificial tendencies through its effect on ideological attachment, defined through the roles of identity fusion with the ingroup and endorsement of violence against outgroups.

### Materials and Methods

#### Participants and Procedure

In Study 2, we aimed to maximize power and so sought to recruit 697 participants via Amazon’s Mechanical Turk ^1^ to achieve greater than 98% power to detect a medium effect of *r* = 0.20 (according to Gignac and Szodorai, 2016, and consistent with the findings of Study 1) at α = 0.001 in our primary analyses of correlations between the cognitive variables and the endorsement of extreme pro-group actions, calculated using the pwr package in R (Champely et al., 2018). We oversampled by 8% and recruited 750 participants, 7 of which were excluded due to providing incomplete responses, yielding a total sample of 743 participants. Participants were recruited from Amazon Mechanical Turk and redirected from these platforms to an online survey hosted by Qualtrics Survey Software for completion of all the self-reported items and the RAT, and later redirected again to Inquisit 5 by Millisecond Software in order to temporarily download software that allows for accurate measure of performance and reaction times in the WCST. Participants were asked about demographic variables such as age, ethnicity, gender, and educational attainment. In the United States sample, the average age was 36.54 (*SD* = 13.45, range = 18 to 81), and 55.4% of participants were female (see Supplementary Table S2 for additional demographic characteristics). The research was conducted with the ethical approval of Cambridge University’s Department of Psychology Research Ethics Committee. The WCST, RAT, and willingness to fight and die for the group were all measured in the same way as in Study 1, except with the latter adapted to the United States sample such that items were focused on the American citizens as the ingroup (e.g., “I would fight someone physically threatening another American person”). Furthermore, when administering the trolley dilemma, participants were presented with the same scenario however, were not asked to indicate their confidence about their decision. An additional cognitive flexibility measure was included (Alternative Uses Test, see below) and an additional measure of ideological attachment was administered (Identity Fusion, see below).

#### Alternative Uses Test (AUT)

In this computerized version of the AUT (Guilford, 1967), two common household items (brick and newspaper) were presented each for 1.5 min. Participants were asked to generate as many possible uses for these items. A timed clock was displayed to participants showing them how much time they had left to complete the task. The responses were analyzed by 4 different measures, in accordance with convention (e.g., Chermahini and Hommel, 2010; Madore et al., 2015; Zmigrod et al., 2015; Addis et al., 2016): (1) *fluency*, the total number of appropriate responses; (2) *flexibility*, the total number of distinct conceptual categories in which the participant’s responses belonged; (3) *elaboration*, the total amount of detail provided for each response (response scored 0 for no details, scored 1 for multiple descriptive words, scored 2 for detailed description of the object use); (4) *originality*, the total number of original responses, defined as responses that appear in less than 5% of the total responses (a response was scored 0 if it was present in more than 5% of the total sample’s responses, and response was scored 1 if present in less than 5% of the sample’s responses). The responses were rated and calculated by two independent raters.

#### Identity Fusion

To measure participants’ feeling of attachment to their nation, participants were presented with a validated measure of identity fusion, the Dynamic Identity Fusion Index (DIFI; Jimenez et al., 2016). The DIFI is a continuous pictorial representation that allows participants to move a small circle representing “the self” by clicking and dragging it toward or away from a large circle representing “the United States of America.” The amount of overlap between the two circles has been shown to indicate the extent to which individuals feel their personal identity is fused with a collective identity (Jimenez et al., 2016). It has temporal stability, as well as convergent and discriminant validity (Jimenez et al., 2016), and has been previously used to measure nationalistic attachment (e.g., Fredman et al., 2017; Zmigrod et al., 2018a).

### Results

#### Replicating H1: Is Cognitive Inflexibility Correlated With Extremist Attitudes?

As in Study 1, performance on the WCST and RAT was analyzed in relation to individuals’ support for extreme pro-group actions. The correlational analysis replicated the significant negative correlations between willingness to fight and kill out-group members to protect the ingroup and cognitive flexibility across both tasks observed in Study 1 (Table 1). The correlations were also consistent for the additional cognitive flexibility task included in Study 2 (the Alternative Uses Test), which was also negatively correlated with support for extreme pro-group actions. The correlations, which are remarkably similar across both samples in Study 1 and 2, show that individuals who were more willing to endorse violence were more cognitively inflexible. Additionally, Study 2 tested for the relationships between cognitive flexibility and nationalistic identity fusion. There was a negative correlation between identity fusion and RAT accuracy rate, but not with WCST or AUT performance.

#### H4: Is Cognitive Inflexibility a *Specific* Cognitive Antecedent to Extremist Attitudes?

In order to test the specificity of cognitive flexibility, relative to other aspects of cognition, in extremist attitudes, a 2-step hierarchical linear regression analysis predicting willingness to fight and kill for the ingroup was conducted (Table 2). Participants who did not provide demographic information or did not perform the AUT were omitted from this analysis, leading to 636 participants. The first step of the regression analysis consisted of demographic variables including gender, educational attainment, and age. In the second step, the four measures that can be extracted from AUT performance were included: Flexibility, Fluency, Elaboration, and Originality. The demographic variables in the first step generated a significant model [*F*(3,632) = 13.686, *p* < 0.001], with gender as a significant predictor of willingness to fight others to protect the ingroup (*p* < 0.001) such that men endorsed extreme pro-group actions to a greater extent than women. The regression model was significantly improved by the AUT measures [Δ*F*(4,628) = 7.187, *p* < 0.001] with the AUT Flexibility serving as the only significant predictor (*p <* 0.001). Overall, the regression model accounted for 10.2% of the variance in willingness to fight for the group. Cognitive flexibility may therefore have a considerable and specific effect on individuals’ susceptibility to outgroup violence endorsement.

**Table 2:** Explaining willingness to fight for the group with performance on the alternative uses task (AUT).

	United States sample		
Variable	B [95% CI LB, UB]	β	*t*
**Step 1**		
Gender	3.371 [2.313, 4.428]	0.242^∗∗∗^	6.259
Educational attainment	–0.370 [-0.772, 0.031]	–0.071	–1.810
Age	0.016 [-0.024, 0.055]	0.031	0.793
	ΔR^2^ = 0.061^∗∗∗^	
**Step 2**			
Gender	3.337 [2.284, 4.389]	0.240^∗∗^	6.224
Educational attainment	–0.332 [-0.730, 0.066]	–0.063	–1.638
Age	0.015 [-0.024, 0.054]	0.029	0.742
AUT flexibility	–0.882 [-1.350, -0.413]	–0.180^∗∗∗^	–3.669
AUT fluency	0.008 [-0.371, 0.386]	0.003	0.039
AUT elaboration	0.127 [-0.121, 0.376]	0.043	1.004
AUT originality	–0.188 [-0.621, 0.244]	–0.060	–0.854
	ΔR^2^ = 0.041^∗∗∗^		
	Overall *R*^2^ = 0.102 Adjusted *R*^2^ = 0.092		
	*N* = 636		
	^∗∗^*p* < 0.01, ^∗∗∗^*p* < 0.001		

*Results of hierarchical linear regression with willingness to fight for the group regressed on educational attainment, age, and gender in the first step, and the four dimensions of the alternative uses task entered in the second step.*

#### H5: Does Cognitive Inflexibility Predict Violence Endorsement Against Outgroups, Identity Fusion, and Self-Sacrificial Tendencies?

As in Study 1, we used structural equation modeling to investigate the extent to which cognitive inflexibility predicts (a) willingness to fight for the group and (b) heightened identity fusion, and whether these in turn predict (c) willingness to sacrifice oneself for the group. We used the R package Lavaan (Rosseel, 2012, 2017) to compute these models. A three-level model was fitted to the data, in which Level 1 included the three cognitive flexibility measures (WCST, RAT, and AUT Flexibility), Level 2 consisted of willingness to fight for the group and nationalistic identity fusion, and Level 3 reflected individuals’ willingness to sacrifice themselves for their national group.

A model was tested in which willingness to self-sacrifice for the group (Level 3) was directly predicted by the ideological attachment variables in Level 2 as well as the cognitive flexibility variables in Level 1. This model had excellent fit to the data [χ^2^ = 18.397, df = 9, *p* = 0.031, *n* = 743, RMSEA = 0.037 (0.010, 0.062), SRMR = 0.024, CFI = 0.981, Yuan-Bentler scaling correction factor = 0.996]. Next, we compared this model, in which all pathways between the cognitive flexibility measures and willingness to self-sacrifice were estimated freely, to a more parsimonious model, in which all pathways between Level 1 and Level 3 were set to equal 0. This parsimonious model reflects the assumption that the effect of cognitive flexibility on willingness to self-sacrifice is mediated completely via the ideological attachment variables in Level 2. This model also had excellent fit to the data [χ^2^ = 22.512, df = 12, *p* = 0.032, *n* = 743, RMSEA = 0.034 (0.009, 0.056), SRMR = 0.026, CFI = 0.978, Yuan-Bentler scaling correction factor = 0.977]. A likelihood ratio test showed no significant difference in model fit between the two models (Δχ^2^ = 4.211, Δdf = 3, *p* = 0.2396), and so the more parsimonious model – which assumes that the ideological attachment variables in Level 2 fully mediate the relationships between Level 1 and 3 – is preferred.

**FIGURE 3:**
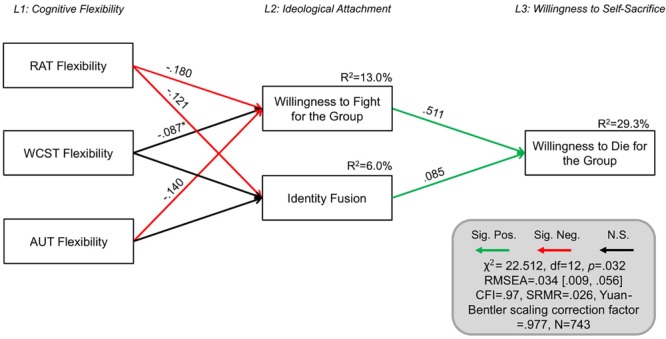
Structural equation model predicting willingness to sacrifice oneself for the group. All parameters shown are fully standardized. For unstandardized estimates, SEs, and confidence intervals, see Supplementary Data. Significant parameter estimates are shown in red and green bolded lines. Residual covariances between variables on the same level are allowed, but not shown for simplicity. Model controls for demographic variables (age, gender, and educational attainment). Significance level was *p* < 0.05. ^∗^*p* = 0.087. L1, level 1; L2, level 2; and L3, level 3. Figure design was inspired by Kievit et al. (2016).

As evident in Figure 3, this model accounted for 29.3% of the variance in willingness to sacrifice oneself for the group (*R*^2^ = 29.0% when not controlling for demographic variables). The model suggests that reduced cognitive flexibility contributes toward heightened ideological attachment, and this in turn predicts a greater willingness to sacrifice oneself to save the life of a fellow American. Out of the three cognitive flexibility measures, RAT negatively predicted nationalistic identity fusion, such that lower scores on the RAT were related to greater identity fusion. Furthermore, RAT and AUT Flexibility were the strongest predictors of support for extreme pro-group actions, and each made significant and complementary contributions to the prediction of willingness to self-sacrifice. While WCST was not a significant predictor of willingness to fight for the group in this model, its contribution approached significance (*p* = 0.087). To probe this further, an SEM was fitted in which the pathway between support for extreme pro-group actions and WCST was estimated freely while the pathways between support for extreme pro-group actions and the other cognitive flexibility measures (RAT and AUT) were constrained to 0. This suggested that WCST is predictive of support for extreme pro-group actions (unstandardized estimate = -8.066, SE = 2.357, standardized estimate = -0.182, *p* = 0.001) but its variance is accounted for by the other cognitive flexibility measures.

Additionally, control analyses were conducted in order to evaluate the models’ hierarchical structure and to establish that the fit of these models was not due to a general feature of the variable covariance matrix. We fitted a model in which Level 1 and Level 2 were reversed, such that willingness to self-sacrifice was predicted by cognitive rigidity, which in turn were predicted by endorsement of extreme pro-group actions and identity fusion. Consequently, this control model included the same information as the original model (depicted in Figure 3), and possessed equivalent complexity, but assumes a different structural relationship between the variables. Pathways between willingness to self-sacrifice and endorsement of extreme pro-group actions and identity fusion were constrained to zero. The original model fit the data significantly better than the inverted model (ΔAIC = 285.764).

**FIGURE 4:**
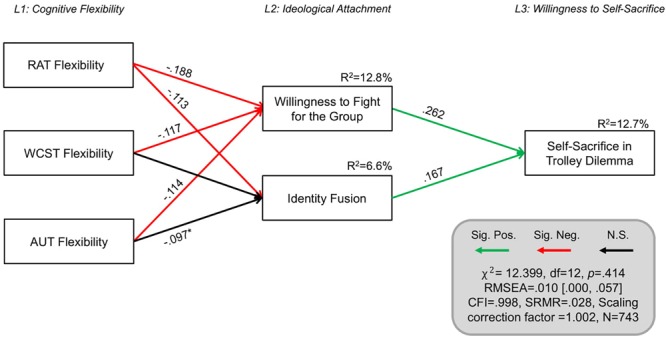
Structural equation model predicting willingness to sacrifice oneself in the trolley problem All parameters shown are fully standardized. For unstandardized estimates, SEs, and confidence intervals, see Supplementary Data. Significant parameter estimates are shown in red and green bolded lines. Residual covariances between variables on the same level are allowed, but not shown for simplicity. Model controls for demographic variables (age, gender, educational attainment). Significance level was *p* < 0.05. ^∗^*p* = 0.062. L1, level 1; L2, level 2; and L3, level 3. Figure design was inspired by Kievit et al. (2016).

To validate and extend this model further, we fitted the original parsimonious model (Figure 4) to participants’ behavior on the trolley problem. Specifically, we examined whether this model structure would predict individuals’ willingness to sacrifice themselves in the trolley problem. To deal with this dichotomous outcome variable, the structural equation model was specified such that diagonally weighted least squares were used to estimate the model parameters, but the full weight matrix was used to compute robust standard errors and mean- and variance-adjusted test statistics (see Rosseel, 2017). This model was an excellent fit to the data [χ^2^ = 12.399, df = 12, *p* = 0.414, *n* = 743, RMSEA = 0.010 (0.000, 0.057), SRMR = 0.028, CFI = 0.998, Scaling correction factor = 1.002, Shift parameter for simple second-order correction = 0.539].

As manifest in Figure 4, this model accounted for 12.7% of the variance in participants’ decision to sacrifice themselves for the group in the trolley problem (*R*^2^ = 12.2% when not controlling for demographic variables). This model suggests that cognitive inflexibility predicts heightened support for extreme pro-group actions and identity fusion, which in turn predict willingness to self-sacrifice in the trolley problem. Notably, all three measures of cognitive flexibility were significant predictors of support for extreme pro-group actions in this model, with each making independent and complementary contributions. With regards to identity fusion, RAT was a significant predictor, and AUT Flexibility’s predictive contribution approached significance (*p* = 0.062). We examined whether a less parsimonious model in which the pathways between Level 1 and Level 3 are estimated freely would have better model fit to the data. While the model fit was also excellent [χ^2^ = 11.749, df = 9, *p* = 0.228, *n* = 743, RMSEA = 0.030 (0.000, 0.073), SRMR = 0.028, CFI = 0.984, Scaling correction factor = 1.043, Shift parameter for simple second-order correction = 0.373], a likelihood ratio test revealed that allowing these pathways to be estimated freely did not significantly improve model fit (Δχ^2^ = 0.0463, Δdf = 3, *p* = 0.9974) and so the more parsimonious model presented in Figure 4 was preferred.

### Interim Discussion

Study 2 replicated the results of Study 1, in a United States participant sample, by corroborating the relationship between willingness to endorse violence against outgroups to protect the ingroup and cognitive inflexibility and (Table 1), supporting *H1*. It also extended the findings of Study 1 by replicating this effect in a third, independent measure of cognitive flexibility, the Alternative Uses Test, and illustrating that cognitive inflexibility was specifically implicated in outgroup violence endorsement, as other cognitive indices regarding verbal fluency, elaboration, and originality, were not implicated (Table 2), supporting *H4*. As in Study 1, WCST performance was not significantly correlated with willingness to self-sacrifice, while the correlation with RAT approached significance (*p* = 0.069), offering some support to Study 1. In contrast, the AUT flexibility score was significantly related to willingness to self-sacrifice (Table 1), suggesting that perhaps different facets of flexibility are differentially related to these psycho-social processes. Lastly, in support of *H2* and *H5*, cognitive inflexibility predicted willingness to fight for one’s ingroup as well as ingroup identity fusion, which jointly predicted self-sacrificial tendencies in self-report measures and the trolley dilemma scenario (Figure 3, 4).

## General Discussion

Rigid ideological thinking and the inflexible categorization of “us” and “them” that underlie a willingness to harm out-group members for the sake of the ingroup were hypothesized to be related to domain-general cognitive rigidity. Across all statistical analyses, in over 1,000 participants from both the United Kingdom (Study 1) and the United States (Study 2), cognitive inflexibility predicted willingness to fight and harm others to protect the national ingroup, which in turn predicted a willingness to die for the group. Indeed, across both samples, structural equation modeling suggested that the relationship between cognitive inflexibility and willingness to self-sacrifice was fully mediated via the role of ideological attachment (Figure 2, 3). These models accounted for 33.5% of the variance in willingness to die for the group in the United Kingdom sample and 29.3% of the variance in the United States sample. Notably, these patterns were consistent when self-sacrificial tendencies were also evaluated with an ingroup trolley dilemma scenario in which participants indicated whether they would jump to their deaths to save five fellow citizens or save themselves (Figure 4). Cognitive inflexibility predicted ideological attachment, which in turn predicted trolley problem sacrifice decisions, accounting for 12.7% of the variance in the binary self-sacrifice decision. In the United Kingdom sample, participants also indicated their level of confidence in their decision to self-sacrifice vs. self-preservation. Greater conviction in the decision to self-sacrifice was related to cognitive inflexibility across both tasks (Figure 1). Moreover, analysis of performance on the AUT revealed that cognitive flexibility – distinctly from other aspects of cognition such as fluency, elaboration, and originality – was specifically implicated as a cognitive antecedent of ideological attachment and self-sacrifice (Table 2).

Humans have a tendency to categorize people into distinct social groups and to enforce rigid boundaries between “us” and “them.” While this simplification is computationally cheap, it also enforces rigidity and can facilitate failures in intergroup empathy that ultimately enable people to inflict harm on outgroups (for review see: Cikara and Van Bavel, 2014). Despite the general tendency toward rigid social categorization, individuals differ in their emphasis on “us” vs. “them” distinctions and consequently in their willingness to harm others and sacrifice themselves in the defense of their ingroup. Here we find that individuals with heightened willingness to support outgroup violence also exhibit heightened cognitive inflexibility in non-ideological, behavioral cognitive tasks. This supports the hypothesis that a general rigidity in evaluation of stimuli translates into a rigidity in assessments of social groups, and this facilitates a proclivity toward ideological outgroup violence endorsement and self-sacrifice. The way in which the mind processes and responds to non-ideological stimuli may therefore reflect the manner in which it evaluates ideological group identities and the permissiveness of inflicting harm on anonymous outgroup members in defense of one’s ingroup.

These findings illustrate that cognitive factors – and not purely emotional or motivational processes – shape endorsement of extreme pro-group actions, such as harming others and self-sacrificing for the group. The results are consistent with other findings that cognitive inflexibility is associated with ideological commitment to conservatism (Van Hiel et al., 2016; Jost, 2017), political extremism (Zmigrod et al., unpublished at JEP:G), nationalism (Zmigrod et al., 2018a), and religion (Zmigrod et al., 2018b). Cognitive inflexibility has also been shown to relate to lack of intellectual humility (the awareness that one’s evaluation of information and decision-making may be biased or flawed; Zmigrod et al., 2019). Cognitive dispositions may therefore need to be incorporated into prominent theories about the factors shaping extremism and self-sacrifice, such as significance quest theory (Kruglanski et al., 2014) identity fusion theory (Swann et al., 2010, 2012), and even the social identity model of collective action (Van Zomeren et al., 2008). This will facilitate research into the neural mechanisms that underlie these psycho-social processes.

An example of how these cognitive findings may inform and complement the neuroscience of intergroup relations and radicalization may reside in the recent findings by Pretus et al. (2018, current Special Issue), showing that neural activity associated with processing of sacred values is heightened in brain regions implicated in rule retrieval amongst individuals susceptible to radicalization. Given that cognitive inflexibility in the Wisconsin Card Sorting Test measured here is operationalized in terms of difficulty in adapting to changes in rules, and left inferior frontal areas have in fact also been implicated in cognitive inflexibility processes (Konishi et al., 1998; Kim et al., 2011), it may be that these neural findings are partially explained by the rigidity of behavioral rules often imposed by sacred values and extremist ideologies. Future empirical work will need to elucidate whether cognitive rigidity and cognitive control processes mediate the relationship between susceptibility to radicalization and neural risk factors.

To our knowledge, this is the first study suggesting that strong ingroup loyalty and sacrifice are not purely underpinned by “hot”emotional processing, attitude-confirming biases, or moral foundations and values. Here it is shown that people’s willingness to harm others for their group may also be rooted in “cold” emotionally neutral cognitive information processing tendencies toward strict categorical thinking and preferences for systematic, persisting rules for thought and behavior. Future research on the psychological roots of radicalization and extremist attitudes will need to address how non-emotional cognitive styles interact with other individual-level motivational risk factors such as the quest for personal significance (Kruglanski et al., 2014), identity fusion (Swann et al., 2010; Whitehouse, 2018), the need to belong (Littman and Paluck, 2015; Lyons-Padilla et al., 2015), social dominance orientation and right-wing authoritarianism (Duckitt and Sibley, 2010), and sacred values (Atran, 2010; Ginges, 2019).

While the present study involved two large samples and a replication of effects, it was constrained to two Western samples, and so further work is needed in other cultural contexts. This is especially important given findings of cultural differences in responses to trolley dilemma problems (Gold et al., 2014a) and the need to make psychological science more demographically and culturally representative (Rad et al., 2018). Additionally, it has been shown that stereotype content shapes endorsement of violence against outgroups and behavior in hypothetical footbridge dilemmas (Cikara et al., 2010) and so it will be essential for future work to incorporate outgroup stereotype content into models of the psychology and neuroscience of radicalization susceptibility. Moreover, we introduced here a novel means of testing conviction in self-sacrifice – by measuring participants’ confidence in their decisions to self-sacrifice in a hypothetical trolley dilemma scenario in order to save the lives of ingroup members. We hope that this will contribute to the methodological discussions surrounding the use of trolley problem dilemmas (e.g., Gold et al., 2014b, 2015; Graham et al., 2016; Cao et al., 2017) and offer a valuable means of subtly testing precursor attitudes toward extremism.

The results are also important due to their implications for future understanding and intervention. Future research will need to investigate the interactions between trait and state cognitive flexibility in relation to endorsement of violence against out-groups; for instance, does strong commitment to an ideological group reduce cognitive flexibility? Developmental studies will be necessary in order to elucidate causal links and self-reinforcing loops between cognitive rigidity and out-group violence endorsement. Moreover, cognitive rigidity may help illuminate the moral logic to reasoning about political violence (Ginges, 2019) and the reasons individuals kill and die for abstract causes. From a prevention perspective, perhaps teaching children and citizens in vulnerable communities to be more cognitively flexible (Diamond and Lee, 2011) would have broader consequences for their tolerance and appetite for conflict than purely targeting their discriminatory attitudes. The finding that cognitive information processing styles are predictive of extremist attitudes should motivate social and political scientists, policymakers, and intervention scientists, to incorporate the objective methodologies of cognitive science in order to construct a more comprehensive picture of the vulnerability factors for extreme and radicalized behavior.

## Ethics Statement

Informed consent was obtained by providing participants with detailed information about the study, its expectations, and their rights, and indicating that their continued participation in this online study will be taken as a marker of their consent but that they will be compensated accordingly if they choose to leave the study at any time. No survey question was compulsory. This study was carried out in accordance with the recommendations of University of Cambridge’s Department of Psychology Research Ethics Committee with written informed consent from all subjects. All subjects gave written informed consent in accordance with the Declaration of Helsinki. The protocol was approved by the Department of Psychology Ethics Committee at the University of Cambridge.

## Author Contributions

All authors contributed to the study design. LZ conducted the data collection, analyzed and interpreted the data, and wrote the manuscript. TWR and PJR provided the critical feedback.

## Conflict of Interest Statement

The authors declare that the research was conducted in the absence of any commercial or financial relationships that could be construed as a potential conflict of interest.

## Footnotes

1 www.mturk.com
