# Supplementary material for: Cognitive Inflexibility Predicts Extremist Attitudes
Source: Front Psychol. 2019 May 7;10:989. doi: 10.3389/fpsyg.2019.00989 (PMC6514156; doi:10.3389/fpsyg.2019.00989)
Supplement: Supplementary file 1 [file Table_1.docx]

**SUPPLEMENTARY INFORMATION**

**Cognitive Flexibility and Demographic Variables**

There were no significant gender differences in cognitive flexibility in both samples. In Study 1, there were no gender differences with regards to the Wisconsin Card Sorting Test, *t*(204)=1.703, *p*=.090, or the Remote Associates Test, *t*(294)=1.456, *p*=.146. Similarly, in Study 2, there were no gender differences with regards to the Wisconsin Card Sorting Test, *t*(396)=-.172, *p*=.863, Remote Associates Test, *t*(639)=1.346, *p*=.179, or Alternative Uses Test Flexibility score, *t*(716)=.416, *p*=.678.

With regards to age, Study 1 found no significant correlations between age and performance on the Wisconsin Card Sorting Test, *r*=-.135, *p*=.057 (approaching significance), or the Remote Associates Test, *r*=-.042, *p*=.480. In Study 2, there were no significant correlations with age in relation to the Wisconsin Card Sorting Test, *r*=-.042, *p*=.403, and AUT Flexibility score, *r*=-.021, *p*=.573. There was a significant correlation in Study 2 between age and RAT, *r*=.136, *p*=.001.

Educational attainment was significantly related to the Remote Associates Test in Study 1, *r*=.175, *p*=.003, and not significantly related to Wisconsin Card Sorting Test, *r*=.118, *p*=.092. Educational attainment was not significantly related to any cognitive flexibility measure in Study 2: the correlation between educational attainment and Wisconsin Card Sorting Test was *r*=.029, *p*=.568, Remote Associates Test, *r*=.061, *p*=.124, and AUT Flexibility score, *r*=.019, *p*=.613.

**Table S1**. Ethnic composition of UK sample (Study 1)

| **Ethnicity - UK Sample** | **Frequency** | **Percent** |
| --- | --- | --- |
| No Response | 4 | 1.32 |
| Arab | 1 | 0.33 |
| Arab & Hispanic/Latino | 1 | 0.33 |
| Asian or Asian British (Indian, Pakistani, Bangladeshi, Chinese, or other) | 8 | 2.63 |
| Black African or Black Caribbean | 2 | 0.66 |
| Other | 5 | 1.64 |
| White | 277 | 91.12 |
| White & Asian or Asian British (Indian, Pakistani, Bangladeshi, Chinese, or other) | 2 | 0.66 |
| White & Black African or Black Caribbean | 3 | 0.99 |
| White & Other | 1 | 0.33 |
| **Total** | **304** | **100** |

**Table S2**. Ethnic composition of US sample (Study 2)

| **Ethnicity - US Sample** | **Frequency** | **Percent** |
| --- | --- | --- |
| No response | 16 | 2.15 |
| American Indian or Alaska Native | 7 | 0.94 |
| American Indian or Alaska Native & Asian & Hispanic/Latino | 1 | 0.13 |
| American Indian or Alaska Native & Hispanic/Latino | 1 | 0.13 |
| Asian | 51 | 6.86 |
| Asian & Hispanic/Latino | 1 | 0.13 |
| Black or African American | 34 | 4.58 |
| Black or African American & American Indian or Alaska Native & Asian & Native Hawaiian or Pacific Islander | 1 | 0.13 |
| Black or African American & Hispanic/Latino | 1 | 0.13 |
| Hispanic/Latino | 19 | 2.56 |
| Native Hawaiian or Pacific Islander | 1 | 0.13 |
| Other | 3 | 0.40 |
| White | 570 | 76.72 |
| White & American Indian or Alaska Native | 8 | 1.08 |
| White & Asian | 12 | 1.62 |
| White & Asian & Other | 1 | 0.13 |
| White & Black or African American | 3 | 0.40 |
| White & Black or African American & American Indian or Alaska Native | 1 | 0.13 |
| White & Black or African American & Hispanic/Latino | 1 | 0.13 |
| White & Hispanic/Latino | 10 | 1.35 |
| White & Other | 1 | 0.13 |
| **Total** | **743** | **100** |
